# Proline‐Selective Electrochemiluminescence Detecting a Single Amino Acid Variation Between A1 and A2 β‐Casein Containing Milks

**DOI:** 10.1002/advs.202411956

**Published:** 2024-12-07

**Authors:** Eunkyoung Kim, Chen‐Yu Chen, Monica J. Chu, Mya F. Hamstra, William E. Bentley, Gregory F. Payne

**Affiliations:** ^1^ Institute for Bioscience and Biotechnology Research University of Maryland College Park Maryland 20742 USA; ^2^ Robert E. Fischell Institute for Biomedical Devices University of Maryland College Park Maryland 20742 USA; ^3^ Fischell Department of Bioengineering University of Maryland College Park Maryland 20742 USA

**Keywords:** β‐Casein, A2 Milk, electrochemiluminescence, proline, single amino acid variation

## Abstract

The proline amino acid and prolyl residues of peptides/proteins confer unique biological and biochemical properties that motivates the development of proline‐selective analysis. The study focuses on one specific class of problem, the detection of single amino acid variants involving proline, and reports a Pro‐selective electrochemiluminescence (ECL) method. To develop this method, the A1‐/A2‐ variants of milk's β‐casein protein are investigated because it is a well‐established example and abundant samples are readily available. Specifically, β‐casein has 209 amino acids with 34 (or 35) proline residues: the A1‐variant has a Pro‐to‐His substitution at position 67 (relative to the A2 variant). The study shows that proline's strong luminescence allows the generic discrimination of: Pro from other amino acids; an A2‐oligopeptide from an A1‐oligopeptide; the A2‐β‐casein variant from the A1‐variant; and commercially‐available A2 milks from A1‐containing regular milks. The evidence indicates that luminescence depends on proline content and accessibility, as well as signal quenching. Compared to conventional immunoassays, the ECL method is simple, rapid, and inexpensive. Further, the ECL‐method is Pro‐selective (vs molecularly‐selective like typical immunoassays) which should make it broadly useful for studying the role of proline in biology and especially useful for tracking the digestion of proline‐rich proteins in the diet.

## Graphical abstract

Proline is important in cellular metabolism, cancer development, and plant stress, while prolyl residues control the structure, interactions, and proteolysis of proteins. Here, a new electrochemiluminescence (ECL) measurement for the generic detection of proline and prolyl residues of peptides/proteins is reported. It is demonstrated that this ECL method can discern milks containing single amino acid variants (Pro‐to‐His) of the β‐casein protein.

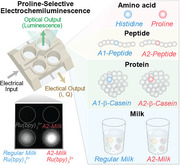

## 1 Introduction

Proline has an unusual cyclic secondary amine structure and this amino acid has been reported to have diverse biological roles^[^ ^1^ ^]^ including: i) a main precursor of extracellular protein (i.e., collagen),^[^ ^2^ ^]^ ii) an energy source for some pathogens,^[^ ^3^ ^]^ iii) an antistress molecule against various insults,^[^ ^4^ ^]^ iv) a neural metabotoxin associated with schizophrenia,^[^ ^5^ ^]^ v) a modulator of cell signaling pathways,^[^ ^6^ ^]^ vi) an inducer of proliferation of stem and tumor cells^[^ ^7^ ^]^ and vii) a modulator of cell morphology and migration/invasiveness.^[^ ^1^, ^8^ ^]^ Increasingly, links are being suggested between proline metabolism and various disease processes (e.g., cancer and diabetes).^[^ ^8^, ^9^ ^]^ In proteins, proline (Pro) residues can dramatically affect: protein conformation (i.e., by disrupting α‐helical structure);^[^ ^10^ ^]^ protein‐protein interactions (e.g., with the proline domain of the p53 protein);^[^ ^11^ ^]^ and proteolytic digestion (e.g., of the proline‐rich gluten proteins).^[^ ^12^ ^]^ While there is growing interest in resolving proline's unique biological activities, one limitation is the absence of simple measurement methods that can detect proline or prolyl residues in complex backgrounds.

One important class of problem is a single amino acid variation involving proline substitutions that significantly alter protein structure and function.^[^ ^13^ ^]^ Examples include proline variation in: collagen that can lead to disorders in bone formation; the p53 tumor suppressor protein that alters DNA binding and potentially leading to cancer; hemoglobin that changes oxygen binding properties; and actin that can affect cell motility by disrupting interactions with other proteins.^[^ ^13^, ^14^ ^]^

**Scheme 1:**
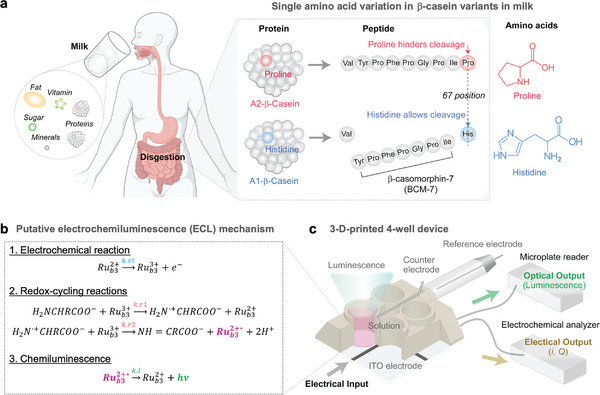
a) Milk can contain either or both variants of β‐casein. The Pro‐to‐His substitution for the A1 variant facilitates proteolysis and formation of a β‐casomorphin‐7 (BCM‐7) bioactive peptide. b) Putative electrochemiluminescence (ECL) mechanisms that show the electrical and optical signals are linked although the linkage across these two modalities is amino‐acid‐dependent. c) Device used to simultaneously measure electrical and optical signals.

Here, we examined a well‐established example of a single amino acid variant involving the Pro‐to‐Histidine (His) substitution at position 67 in the β‐casein milk protein.^[^ ^15^ ^]^ Of the 209 amino acids in this protein, 35 are prolyl residues for the A2 variant and 34 for the A1 variant.^[^ ^16^ ^]^ This single amino acid substitution significantly affects casein's structure^[^ ^15^, ^16^, ^17^ ^]^ and digestion.^[^ ^15b–d^ ^]^ As illustrated in **Scheme** **1**a, the His67‐containing A1 variant (but not the Pro67‐containing A2 variant) is digested in the gastrointestinal tract leading to the generation of β‐casomorphin‐7 (BCM‐7) which is a bioactive opioid peptide reported to result in adverse responses in the gastrointestinal, cardiovascular, neurological and endocrine systems.^[^ ^15a,c,d^ ^]^ Because the A2 milk is considered by some to be a healthier alternative to A1 milk,^[^ ^15^, ^18^ ^]^ various advanced analytical detection methods to differentiate one from the other have been developed (e.g., genotyping^[^ ^12^ ^]^ and immunoassays^[^ ^19^ ^]^). In this study, we used the single amino acid variation of casein as our example because samples (i.e., milks) are abundant, inexpensive, and available without the need for special precautions or approvals, thus this example allows the extensive development of the ECL method.

Specifically, we report a rapid (≈ 20 min) electrochemiluminesence (ECL) method that offers high selectivity for proline and prolyl residues of proteins, and we demonstrate that this method can discern commercially‐available A2 milk from regular milk based on the higher ECL levels of the A2 β‐casein variant. In principle, ECL could combine the speed and simplicity of electrochemistry with the benefits of chemiluminescence (i.e., low background, high sensitivity and wide dynamic range).^[^ ^20^ ^]^ While typical ECL methods (e.g., for immunoassay detection) employ washing steps to remove interferents and require the addition of both a luminophore‐label and co‐reactant for signal generation,^[^ ^20^, ^21^ ^]^ we report a direct detection method in which the luminophore Ru(bpy)_3_^2+^ (Ru_b3_^2+^) is added directly to a complex sample matrix.^[^ ^21^, ^22^ ^]^

Although the ECL mechanism is not completely understood,^[^ ^21^, ^23^ ^]^ Scheme 1b shows a putative ECL mechanism proposed in previous studies.^[^ ^22^, ^24^ ^]^ First, Ru(bpy)_3_^2+^ is electrochemically oxidized when an anodic potential is applied. Second, the oxidized Ru(bpy)_3_^3+^ undergoes two redox‐cycling reactions with amino acids and in one case can be reduced to excited Ru(bpy)_3_^2+*^. Redox‐cycling leads to an amplification of the electrical signal (i.e., Ru(bpy)_3_^2+^ oxidation currents). And then, the excited Ru(bpy)_3_^2+*^ relaxes to the ground state (Ru(bpy)_3_^2+^) with the generation of an optical signal (i.e., emission of luminescence). We report that the linkage between these electrical and optical signals varies with the amino acid type, and that proline has a unique ECL response that enables discrimination of the added prolyl residue of the A2 milk. For our measurements, we used a previously‐described 3D printed 4‐well device illustrated in Scheme 1c.^[^ ^25^ ^]^ The base of each well has a transparent indium tin oxide (ITO) electrode that allows electrical inputs to be imposed (e.g., an oscillating potential, *E*) and electrical outputs (i.e., current, *i* and charge, *Q* = ∫*i*d*t*) to be measured. The device was placed in a standard well‐plate reader to allow the simultaneous measurement of these electrical and optical (i.e., luminescence) signals.

## 2 Results and Discussion

### 2.1 Cluster Analysis of the Response of Amino Acids

**Figure 1:**
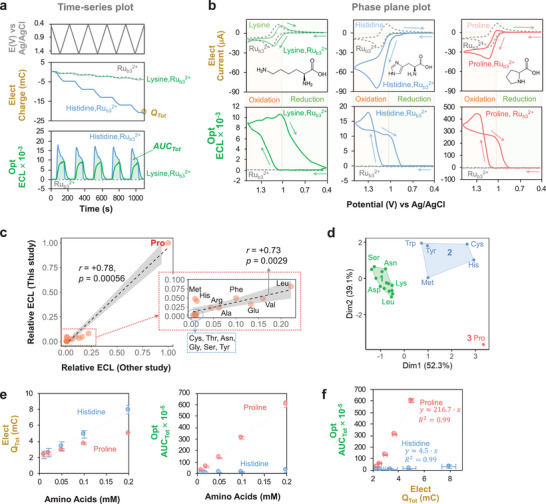
Proline has a unique ECL response compared to other amino acids. a) Time series plots of input (*E*) and outputs (electrical, *Q* = ∫*i*d*t*; and optical, *ECL*) for 5‐cycle cyclic voltammogram (CV). Lys and His have different response‐patterns and quantitative features (*Q_Tot_* (Total Charge) and *AUC_Tot_* (Total Area Under Curve)). b) Phase plane analysis shows: Lys has a small electrical response (comparable to the Ru(bpy)_3_^2+^ control); His has a strong electrical (i.e., oxidative) response; and Pro has an intermediate electrical response but a very strong optical response (ECL). c) Our relative ECL responses for 15 amino acids compares with previous measurements^[^ ^22a^ ^]^ using Spearman's correlation coefficients (r). A gray overlay indicates 95% confidence band for the best‐fit linear regression line (dotted line). d) Cluster analysis shows that Pro forms its own group (silhouette coefficient = 0.57). e) Electrical and optical responses vary linearly with amino acid concentration in an amino acid‐dependent manner. f) Cross‐modal analysis for His and Pro that differ between A1 and A2 β ‐casein variants (the dotted lines represent the fitted linear regression lines). All data are shown as the mean or the mean with the error bar presenting ± standard deviation (N = 4).

In initial studies, we examined the ECL response of 20 individual amino acids (1 mm in phosphate buffered saline (PBS); pH 7.4) to the Ru(bpy)_3_^2+^ luminophore (1 mm) and a cyclically‐imposed potential (*E*) input from 0.4 to 1.5 V (vs Ag/AgCl; scan rate 10 mVs^−1^). **Figure** **1**a shows the five‐cycle time series input‐output curves for two representative amino acids (Lysine, Lys and His) that show markedly different responses (a control containing Ru(bpy)_3_^2+^ without amino acids is also shown). These results show that an oscillating imposed *E* induces: i) a slight oscillating *Q* response for Lys (and the Ru(bpy)_3_^2+^ control), but a monotonous decrease in *Q* for His; and ii) an oscillating optical response for both Lys and His although the pattern (i.e., shape of output curve) differed between these amino acids.

The differences in the ECL‐response are further revealed from phase‐plane analysis. Figure 1b shows electrical (*i‐E*) and optical (*ECL‐E*) phase‐plane plots for Lys, His, and Pro analysis (Note: Figure S1, Supporting Information, provides phase‐plane plots of all amino acids). For Lys, the observed electrical response is similar to that of the Ru(bpy)_3_^2+^ control that indicates that Lys does not undergo electrochemical reactions or redox‐cycling reactions with Ru(bpy)_3_^3+^. In contrast, the electrical response for His and Pro shows amplified oxidation currents and attenuated reducing currents (relative to the Ru(bpy)_3_^2+^ control) that indicates significant redox‐cycling with these amino acids as illustrated in Scheme 1b. Presumably, the differences in redox‐cycling also influence the production of the excited Ru(bpy)_3_^2+*^, yielding a small optical response for Lys and considerably larger optical responses for both His and Pro. Importantly, while the shape of the optical (*ECL‐E*) pattern for His and Pro look similar in Figure 1b, the intensity of the luminescence is 20‐fold higher for Pro.

Previous investigators have observed different ECL responses for various the amino acids.^[^ ^22^, ^24^ ^]^ Figure 1c shows that the relative ECL responses measured in the present study are strongly correlated (r = +0.78, p = 5.6×10^−4^) to those reported in earlier studies^[^ ^22a^ ^]^ (Note: Figure S2, Supporting Information, provides the values of relative ECL). The trends in Figure 1c, show: i) the secondary amine, proline, has the strongest ECL (> 10‐fold higher compared to the other, primary, amino acids); ii) electron‐donating substituents at the α‐carbon atom (e.g., for Leu, Val) tend to enhance ECL, while electron‐withdrawing substituents (e.g., for Ser, Thr) tend to diminish the ECL; and iii) aromatic substituents (e.g., for Tyr, Trp) tend to quench ECL. These trends support the hypothesis that the ECL strength increases with the amino acid's reducing ability which can stabilize the amino acid radical cation and promote the production of the excited Ru(bpy)_3_^2+*^ that is responsible for luminescence.^[^ ^22^, ^24^ ^]^

Next, we performed k‐means cluster analysis^[^ ^26^ ^]^ to discern the differences in ECL‐response for these amino acids. For this analysis, we used four signal features: i) the cumulative charge, *Q_Tot_*, for the five‐cycle time series (illustrated in Figure 1a); ii) the cumulative area under the luminescence curve, *AUC_Tot_*, for the five‐cycle time series (also illustrated in Figure 1a); iii) log(*Q_Tot_*); and (iv) log(*AUC_Tot_*). [Figure S3, Supporting Information, provides the electrical and optical signal metrics (i.e., features) of all amino acids and compares the plots from cluster analysis to cross‐modal (optical vs electrical) plots for these 20 amino acids.] The results in Figure 1d show three clusters (silhouette coefficient = 0.57; reasonable clustering):^[^ ^26^ ^]^ the largest cluster (14 amino acids; shown in green) had small electrical responses consistent with an absence of redox‐cycling (e.g., Lys); another cluster (5 amino acids; shown in blue) had significant redox‐cycling as illustrated by an amplified oxidation response (e.g., His); and a final cluster of a single amino acid (Pro) had a very large luminescence response (∼90 times higher than the average ECL response of other amino acids).

As mentioned, the A2 and A1 β‐casein variants have a single Pro‐His substitution, so we next examined the ECL response to these two individual amino acids by performing five‐cycle input–output experiments at different concentrations (Figure S4, Supporting Information, shows the time series curves for these experiments). Figure 1e shows that for each amino acid, the electrical and optical output metrics (*Q_Tot_* and *AUC_Tot_*) increased linearly with concentration, while Pro showed a weaker electrical response but far stronger optical response (vs His). The limits of detection (LOD) were estimated from an *AUC_Tot_* standard curve in the low concentration region (0.1−10 µm) to be 75 nm (11 pmol) for Pro and 1196 nm (179 pmol) for His (Note: Figure S4, Supporting Information, provides details of LOD calculation). The cross modal optical‐electrical plot (*AUC_Tot_* vs *Q_Tot_*) in Figure 1f shows a nearly 50‐fold difference between Pro and His.

Overall, the results in Figure 1 indicate that Pro has a unique response (especially luminescence response) in our ECL measurement compared to the other 19 amino acids.

### 2.2 Discerning A1 from A2 Peptides

**Figure 2:**
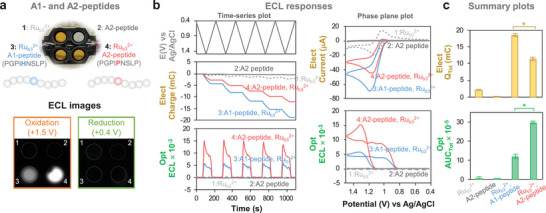
Selectivity of ECL for a 9‐amino acid peptide with a single amino acid variation (Pro‐to‐His). a) Illustration of the peptides tested and the ECL method. b) Time series input‐output plots and phase plane plots for the His‐containing A1 and Pro‐containing A2 peptides. c) Summary plot of electrical and optical responses. For all bar graphs, all data are shown as the mean with the error bar representing ± standard deviation (N = 4). *p* values were calculated with the Kruskal‐Wallis test and the statistical significance is defined as: * = p < 0.05.

Next, we examined proline‐selectivity when the proline residue is localized in a peptide. Specifically, we examined synthetic oligopeptides with the amino acid sequence for residues 63 through 71 for the two β‐casein variants^[^ ^19^ ^]^ (Figure S5, Supporting Information, shows the mass spectra of two synthesized oligopeptides). As illustrated in **Figure** **2**a, these peptides included the single amino acid variation at position 67 for the A1 and A2 variants, and these peptides are both rich in Pro (3 of 9 for the A1 peptide, and 4 of 9 for the A2 peptide). To illustrate the ECL response, we filled the 4 wells with different combinations of peptide (2 mm) and the Ru(bpy)_3_^2+^ luminophore (1 mm) as shown by the photograph in Figure 2a. In the luminescence images in Figure 2a, upon imposing an oxidative potential (+1.5 V for 2 min), the two bottom wells (containing both peptide and the Ru(bpy)_3_^2+^ luminophore) showed luminescence while the response for the A2 peptide was considerably stronger. After imposing a reducing potential (+0.4 V for 2 min), Figure 2a shows that the luminescence disappeared. Figure S6 and Movie S1 (Supporting Information) provide these ECL images and their animation for five‐repeated oxidation and reduction step potentials. To provide a more quantitative analysis for these peptides, we performed five‐cycle time series experiments and performed phase‐plane analysis (Figure 2b). Figure 2c summarizes these results by showing the A2 peptide has a weaker electrical response and a stronger optical response (compared to the A1 peptide).

**Figure 3:**
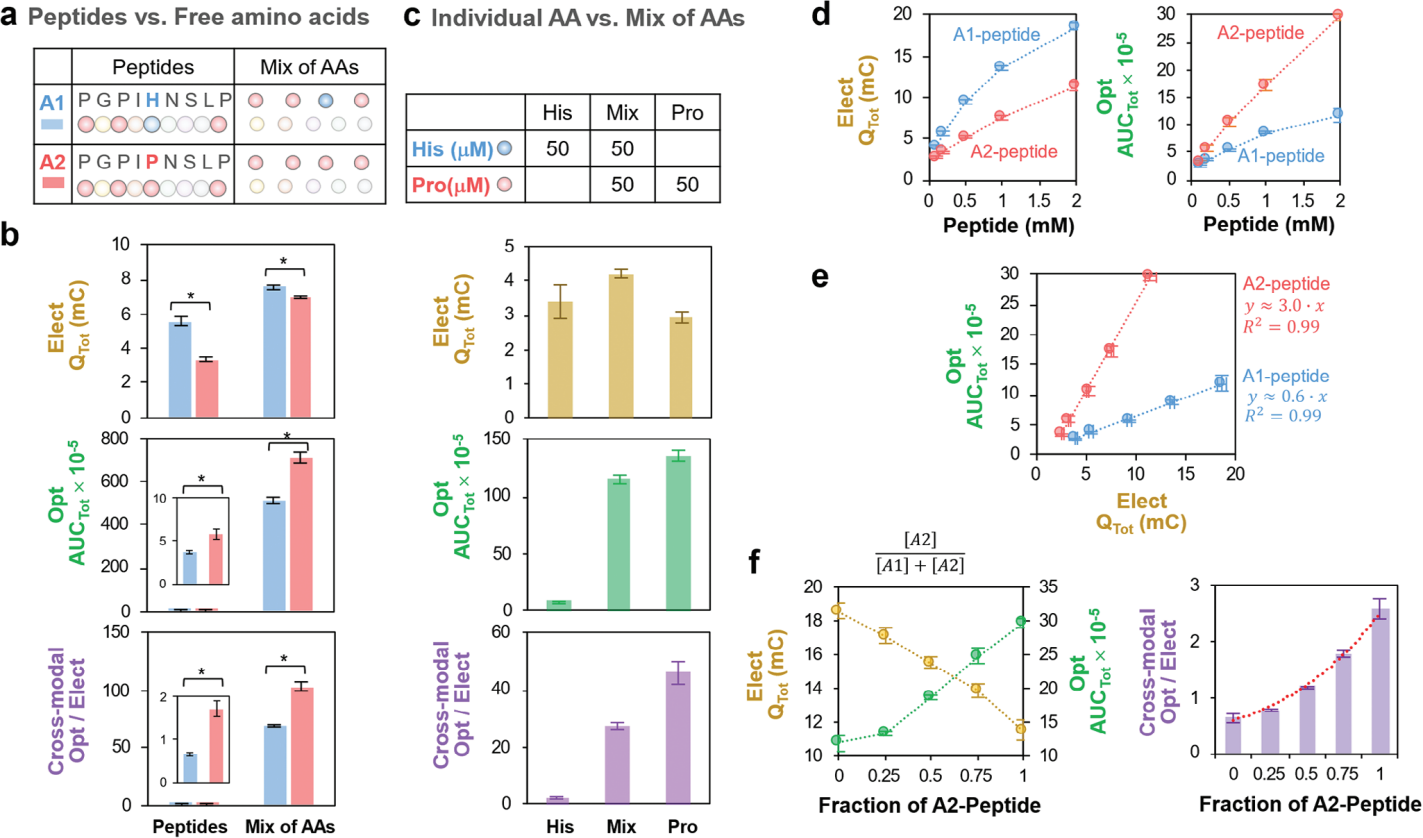
Evidence that quenching is important in the observed ECL response. a) Comparison of peptides to an equivalent mixture of amino acids. b) The optical (ECL) and cross‐modal response of peptides is attenuated compared to the amino acids. *p* values were calculated with the Kruskal–Wallis test and the statistical significance is defined as: * = p < 0.05. c) Comparison of the response of His‐only and Pro‐only to the response of a His‐Pro mixture. The optical (ECL) and cross‐modal response shows that His attenuates the Pro response. d) Electrical and optical responses vary with peptide concentration. e) Cross‐modal analysis for the A2 and A1 peptides. The dotted lines represent the fitted linear regression lines. f) The quantitative signal metrics (*Q_Tot_* and *AUC_Tot_*) and cross‐modal metric vary with the relative amounts of the A1 and A2 peptides: the nonlinearity suggests the A1 peptide may quench the A2‐peptide's ECL response. A red dotted curve represents the fitted exponetial regression curve. All data are shown as the mean with the error bar representing ± standard deviation (N = 4).

There are two observations that indicate that the observed ECL response is not simply dependent on proline content. First, the optical response (*AUC_Tot_*) for the free amino acids (Figure 1e) is significantly higher (>100‐fold) than that for the peptides (Figure 2c). To further investigate this observation, **Figure** **3**a illustrates that we compared ECL measurements of A1‐ and A2‐peptide solutions (0.2 mm) to measurements of solutions prepared from free amino acids of equivalent composition and concentration. The electrical response metric (*Q_Tot_*) in Figure 3b shows comparatively small (although statistically significant) differences between the free amino acids and peptides. In contrast, the optical response metric (*AUC_Tot_*) in Figure 3b shows that the free amino acids have vastly higher (100‐fold) luminescence than the peptides (as expected, higher responses were observed for the peptide or amino acid solutions with higher Pro content). The attenuated ECL‐response for the peptide (vs amino acid mixture) suggests that the localized amino‐acid residues in the peptide quench the ECL response.

The second observation to indicate that the ECL response is not simply dependent on proline content is the observation that despite the A2‐peptide having a 1.3‐fold higher Pro content (vs A1‐peptide), the cross‐modal metric of A2‐peptide in Figure 3c is 2.6‐fold higher than that of A1‐peptide. To examine this effect, Figure 3c compares the ECL response for amino acid solutions containing histidine (50 µm), proline (50 µm), and an equimolar mixture of proline and histidine (50 µM each). Despite doubling the amino acid concentration in the mixture, Figure 3c shows that the ECL response decreased, which suggests that histidine quenches proline's ECL response.

We also performed experiments with different concentrations of the A1 or A2 peptides. The time series input‐output curves are shown in the Supporting Information (Figure S7, Supporting Information) while the electrical response and optical responses are shown in Figure 3d. The cross modal optical‐electrical plot (*AUC_Tot_* vs *Q_Tot_*) in Figure 3e shows a nearly five‐fold difference between A2 and A1 peptides.

In a final experiment, we prepared a mixed solution of A1 and A2 peptides (total peptide concentration of 2 mm) and performed multi‐cycle cyclic voltammetry measurements (Figure S8, Supporting Information, shows the time‐series input‐output curves). Figure 3f shows that as the fraction of A2 peptide increased in the mixture, the electrical output (*Q_Tot_*) decreased by ≈40% while the optical output (*AUC_Tot_*) increased nearly three‐fold. Figure 3f shows that the cross‐modal metric increases with the A2‐peptide fraction (i.e., proline‐content) in a non‐linear manner. This non‐linearity suggests that ECL signal from the A2‐peptide is partially quenched by the A1‐peptide.

In summary, the results of assaying peptides: i) demonstrate that our ECL measurement can discern a single Pro‐His substitution in a 9 amino acid peptide despite this peptide's comparatively high Pro content; and ii) suggest that the ECL signal of proline residue can be quenched by the localized amino‐acid residues in the peptide, which may contribute to the discerning capability of single amino acid variation in peptide. In addition, the results in Figure 3 indicate that the proposed reaction mechanisms in Scheme 1b are incomplete, and that when ECL is performed in interfering backgrounds, quenching effects may attenuate the observed responses. Further mechanistic studies will be required to understand these quenching effects.

### 2.3 Discerning the A2 from A1 β‐Casein Protein Variants

**Figure 4:**
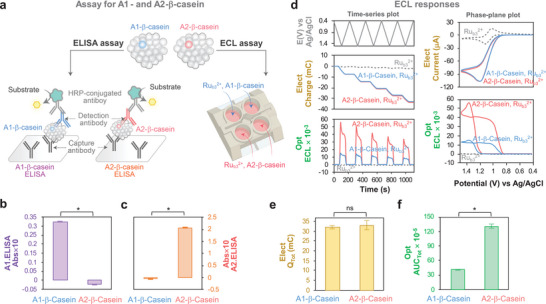
ELISA test kits and ECL can distinguish the protein standards for the A1 and A2 β‐casein variants. a) Schematic of ELISA and ECL measurements. b) As expected, ELISA kit designed to detect the A1‐variant shows low response to the A2‐variant. c) As expected, ELISA kit designed to detect the A2‐variant shows low response to the A1‐variant. All ELISA data are shown as the mean with the error bar representing ± standard deviation (N = 4). d) Time series input‐output plots and phase plane plots for the A1‐ and A2‐β‐casein standards. e) The electrical response metric (*Q_Tot_*) cannot distinguish the A1‐ and A2‐β‐caseins. f) The optical response metric (*AUC_Tot_*) can distinguish the A1‐ and A2‐β‐caseins. All ECL data are shown as the mean with the error bar representing ± standard deviation (N = 3). All *p* values in bar graphs were calculated with the Kruskal–Wallis test and the statistical significance is defined as: * = p < 0.05, ns (not siginificant) = p > 0.05.

Next, we examined ECL's ability to distinguish the single amino acid variation between two casein variants possessing the complex structure of 209 amino acids (vs 9 amino acids in peptide). Because of the interest in distinguishing A2‐ from A1‐milk, ELISA assay kits are commcerically available for both the A2 and A1 β‐casein protein variants as illustrated in **Figure** **4**a. As expected, when 10 ng mL^−1^ of the A1 β‐casein protein standard was tested in the A1‐ELISA assay, Figure 4b shows that a high response was detected; however, when this A1 protein standard was tested in the A2‐ELISA assay, Figure 4c shows that a low signal was detected. Conversely as expected, when 10 ng mL^−1^ of the A2 β‐casein protein standard was tested in the A1‐ELISA assay, Figure 4b shows that a low response was detected; however, when this A2 protein standard was tested in the A2‐ELISA assay, Figure 4c shows that a high signal was detected. This result confirms that the two ELISA assay kits could selectively distinguish the A1‐ and A2‐ β‐casein variants.

The schematic in Figure 4a shows that we used the same two β‐casein protein standards (200 ng mL^−1^) to evaluate the selectivity of our ECL measurement for discerning the A2‐β‐casein from the A1‐β‐casein. The time‐series input‐output plots and phase‐plane plots in Figure 4d show that the A1 and A2 caseins have similar electrical output responses, while the A2 casein has a much higher optical response (vs the A1 casein). In summary, Figure 4e shows there is no significant difference in the electrical responses (*Q_Tot_*) between the A1 and A2 casein variants, while Figure 4f shows significantly different optical responses between these variants.

### 2.4 Discerning the A2 Milk from Regular Milk

**Figure 5:**
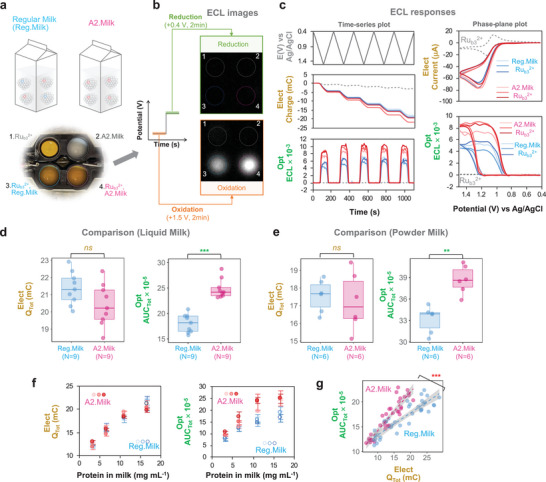
ECL can distinguish regular milks from A2 milks. a) Illustration of experimental approach. b) Images showing a stronger ECL‐response for A2 milk (vs regular milk). c) Time series input‐output plots and phase plane plots for the regular and A2 milks. d) The electrical response shows little discriminating ability, while significant differences were observed in the optical response for the regular and A2 liquid milks. e) Consistent responses were observed for the regular and A2 powder milks. For all bar graphs, *p* values were calculated with the Kruskal–Wallis test and the statistical significance is defined as: ** = p < 0.005, *** = p < 0.0005, ns = p > 0.05. f) Electrical and optical responses as a function of milk protein (i.e., with dilution of the milks). All data are shown as the mean with the error presenting ± standard deviation (N = 3). g) Cross‐modal analysis for regular and A2 milks. The gray overlays indicate 95% confidence bands for the best‐fit linear regression line (dotted line). *p*‐value between the different slopes of two milks was calculated using a multiple linear regression model and the statistical significance is defined as: *** = p < 0.0005.

Finally, we examined the ability of the ECL method to discern the single amino acid variation of the casien proteins in the complex milk background. For this analysis, we purchased 3 different brands of A2 milk and 3 different brands of regular milk (note regular milk is expected to contain a mixture of A2‐ and A1‐ β‐caseins). Figure S9 (Supporting Information) provides the nutrition facts for all milks and illustrates the complexity and potential interferents in milk. As a simple illustration, **Figure** **5**a shows a photograph of 4 wells that were filled with different combinations of 2‐fold diluted milk and the Ru(bpy)_3_^2+^ luminophore (1 mm). The luminescence images in Figure 5b show that upon imposing an oxidative potential (+1.5 V for 2 min) the two bottom wells (containing both milk and the Ru(bpy)_3_^2+^ luminophore) showed: a luminescence response for both milks; and the luminescence response for the A2 milk was considerably stronger than for the regular milk. After imposing a reducing potential (+0.4 V for 2 min), Figure 5b shows that the luminescence disappeared. Figure S10 and Movie S2 (Supporting Information) in the Supporting Information provide these ECL images and their animation for five‐repeated oxidation and reduction experiments.

To provide a more quantitative analysis for these milks, we performed five‐cycle time series experiments and performed phase‐plane analysis. The plots in Figure 5c show results for three A2 milks and three regular milks containing the same protein content (after the two‐fold dilution the total milk protein is estimated to be 16 mg mL^−1^. The box plots in Figure 5d compare the electrical (*Q_Tot_*) and optical responses (*AUC_Tot_*) for triplicate measurements of the regular milks (N = 9) and A2 milks (N = 9). Small, not statistically significant, differences were observed in the electrical responses between the regular and A2 milks, while larger, statistically significant differences were observed in their optical responses.

To expand the applicability of this method from liquid milk, we assayed in triplicate one Regular and one A2 powder milk that is commercially available. For this assay, we prepared powder milk solution by dissolving the milk powder in warm water according to each company's instructions. We then diluted each powder milk solution with a buffer solution to obtain the same protein concentration as liquid milk. As with liquid milk, Figure 5e shows that the optical response of A2 powder milk is higher than that of regular power milk and the difference is statistically significant (Note: Figure S11, Supporting Information, provides the nutrition facts of powder milk).

We also investigated ECL responses dependent on the total milk protein by changing the dilution of liquid milk (Note: An initial total milk protein concentration was obtained from the nutrition facts provided on the milk's label). The time series output plots for these ECL experiments are provided in Figure S12 (Supporting Information). Figure 5f shows nearly linear responses for both the electrical and optical responses with respect to the total milk protein. Consistent with the results in Figure 5d the electrical responses are similar for the two types of milk while the A2 milks show higher optical responses (compared to the regular milks). Also, Figure S13 (Supporting Information) shows that the ECL response can statistically‐significantly discriminate A2 milk from regular milk in all ranges of milk protein concentration (3–33 mg mL^−1^) and the LOD of the discerning ability is estimated from the statistical analysis to be ≈3.3 mg mL^−1^ milk protein (p = 6.7×10^−4^). The cross modal optical‐electrical plot (*AUC_Tot_* vs *Q_Tot_*) in Figure 5g shows a nearly two‐fold difference between the A2 and regular milks. Also, the statistical analysis of this cross‐modal plot using a multiple linear regression model^[^ ^27^ ^]^ supports that the slope difference between regular milk and A2 milk is statistically significant (p = 6.57×10^−5^) (Note: Figure S12, Supporting Information, provides the statistical analysis results).

**Figure 6:**
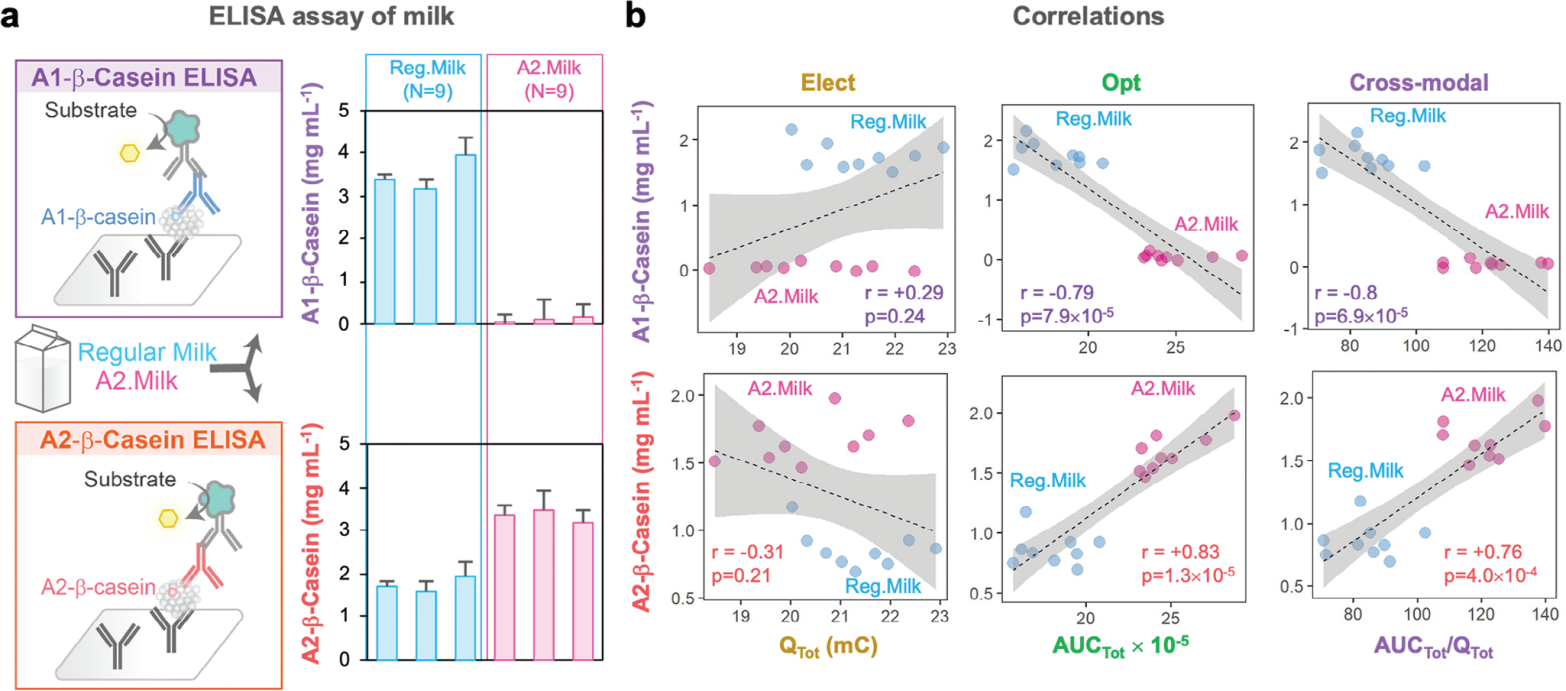
Validation of the ECL method by comparison with immunoanalysis. a) Immunoassays with A1‐specific and A2‐specific ELISAs show that the A2 milk has only the A2 β‐casein variant while regular milk has a mixture of A1 and A2 variants. Each bar is shown as the mean with the error presenting standard deviation (N = 3 for each milk). b) The correlations between the ELISA and ECL method indicate that the optical metric (*AUC_Tot_*) and cross‐modal metric (*AUC_Tot_* / *Q_Tot_*) distinguish regular and A2 milks based on their levels of their β‐casein variants (the electrical metric, *Q_Tot_*, by itself cannot discriminate regular and A2 milks). Spearman's correlation coefficients (r) are indicated. All gray overlays indicate 95% confidence bands for the best‐fit linear regression line (dotted line).

We next compared our ECL method with the ELISA methods for discriminating A2 and regular milks. **Figure** **6**a shows that when the ELISA assay that is specific for the A1‐β‐casein protein was performed with regular milks (N = 9), 3–4 mg mL^−1^ of the A1 variant was detected. As expected, when the A1‐specific ELISA assay was performed with A2 milks (N = 9), little of the A1 variant was detected. When the A2‐specific assay was performed, the regular milks showed about 1.5–2 mg mL^−1^ of the A2 variant, while the A2 milks showed 3–4 mg mL^−1^ of the A2 β‐casein variant. These results are expected since the A2 milk is expected to contain exclusively the A2‐ β‐casein variant while the regular milk contains a mixture of the A1 and A2 variants.

The ELISA measurements for these milks were compared with the ECL measurements for these same milk samples (after a 2‐fold dilution). The y‐axes in Figure 6b are for the ELISA assays, and all 6 plots show that the red circles (A2 milk) and blue circles (regular milk) can be distinguished from each other based on their different y‐values. In contrast, the x‐axis for the plots in the first column of Figure 6b is the electrical response metric (*Q_Tot_*) and the red circles (A2 milk) and blue circles (regular milk) cannot be distinguished from each other based on their x‐values (i.e., the electrical response cannot distinguish the A2 from regular milks). As a result, there is no statistically significant correlation between ELISA results and the electrical responses of the ECL measurement (p > 0.05). The two plots in the central column of Figure 6b have an x‐axis of the optical response metric (*AUC_Tot_*). The red circles (A2 milk) and blue circles (regular milk) can be distinguished based on their optical responses. Also, while the upper plot shows that A1‐β‐casein level of ELISA has a statistically significant negative correlation with the optical response (r = −0.79, p = 7.9×10^−5^), the lower plot shows that A2‐β‐casein of ELISA has a statistically significant positive correlation with the optical response (r = +0.83, p = 1.3×10^−5^). The final column of plots in Figure 6b is for a composite, cross‐modal, feature metric (*AUC_Tot_* / *Q_Tot_*). Like the optical response, the cross‐modal metric can also discriminate A2 from regular milk and shows statistically significant correlations for the A1‐ (r = −0.8, p = 6.9×10^−5^) and A2‐β caseins (r = +0.76, p = 4.0 ×10^−5^) with the ELISA assays. These results show that the ECL and immunoassay methods are correlated, and the ECL method can discriminate regular from A2‐milk based on differences in the β casein variant.

### 2.5 Proline‐Selective and Generic Nature of ECL Method

**Figure 7:**
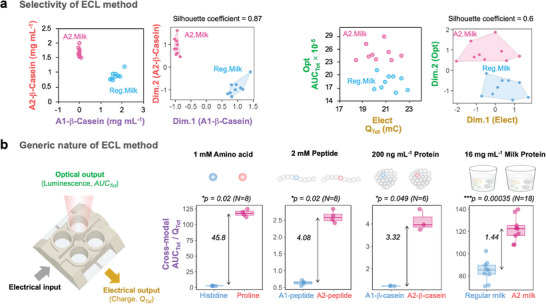
Comparison of the ECL method with conventional methods. a) The selectivity of the ECL is compared to gold standard immunoassays: data and Cluster analysis show that both the ELISA and ECL methods can distinguish A2 from regular milks (Silhouette coefficient = 0.87 (immunoassay); 0.6 (ECL)). b) The generic nature of the ECL method is illustrated by the cross‐modal metric (*AUC_Tot_* / *Q_Tot_*): this metric decreases as the analysis becomes more challenging (from distinguishing amino acids, to distinguishing proline‐rich peptides and proteins, and samples in complex backgrounds) yet the ECL method can discern the A2 and regular milks. *p* values were calculated with the Kruskal–Wallis test.

Compared with other conventional methods, there are two important capabilities of the ECL method. First, the proline‐selectivity of the ECL method is capable of distinguishing milk samples based on their differences in the β casein variants and this is analogous to the capabilities of “gold standard” immunoassays. The first plot in **Figure** **7**a shows the data from the A2‐immunoassay (y‐axis) and A1‐immunoassay (x‐axis) for the 9 measurements of regular milk (blue) and 9 measurements of A2 milk (red). Cluster analysis of this immunoassay data is shown in the second plot of Figure 7a (Silhouette coefficient = 0.87; strong clustering). As expected, both plots show that immunoassays can easily discriminate the A2 and regular milks. The third plot in Figure 7a shows a cross‐plot of the experimentally‐measured optical (*AUC_Tot_*) and electrical feature metrics (*Q_Tot_*) from the ECL measurements, while the final plot shows Cluster analysis for this data (Silhouette coefficient = 0.6; reasonable clustering). Both plots demonstrate that the ECL method can distinguish the regular and A2 milks. While it is not surprising that the molecular‐recognition capabilities of an immunoassay can distinguish a single amino acid variant, it seems surprising to us that the Pro‐residue selectivity of the ECL method can distinguish the A1 variant (a total of 34 Pro residues) from the A2 variant (a total of 35 Pro residues) and this discrimination occurs in a complex matrix (i.e., milk).

A second important capability of this ECL method is that its selectivity appears to be generic for proline and prolyl residues of peptides and proteins. To examine the generic nature of ECL method, a cross‐modal metric is used because it provides a quantitative means to “track” the ECL responses of a single amino acid variation across various systems. Figure 7b shows that the cross‐modal feature metric (*AUC_Tot_* / *Q_Tot_*) has high distinguishing capabilities for amino acids (e.g., Pro from His) and becomes progressively less discerning as the proline‐containing entity becomes more complex (e.g., a high Pro‐content peptide or protein) and when the matrix becomes more complex (e.g., from buffer solution to milk). The decrease in discerning power in more complex matrices might be due to quenching effects by interferents in the matrix (e.g., amino acids, fats and other components) and a decreased accessibility of proline or prolyl residue to Ru(bpy)_3_^2+^ luminophore. Despite this diminishing discerning ability, the ECL method could still distinguish A2 from regular milk. For comparison, Figure S14 (Supporting Information) shows that a colorimetric proline assay can easily distinguish proline from histidine, but cannot distinguish the A1 from A2 peptides, or regular from A2 milks. This indicates that the conventional proline method requires a sample processing step to separate proline from interferences in the background matrix, whereas our ECL method directly measures proline or prolyl residues in complex samples.

The potential importance of a generic proline‐selective ECL assay is that there are a growing number of examples where proline or proline‐rich peptides and proteins are proposed to possess important biological activities and thus a generic proline‐selective assay may have broad applications.

## 3 Conclusion

Here, we report an electrochemiluminescence (ECL) method to detect the amino acid proline and prolyl residues of peptides and proteins. We specifically focused on the discrimination of proteins with a single proline substitution: several examples show that such proline substitutions can disrupt protein structure, biological activity, and digestion. As our experimental example, we evaluated the descrimination of milks containing different variants of the β‐casein protein (the A1 variant has a Pro‐to‐His substitution at position 67 relative to the A2 variant). This example allows: methods‐development using samples (i.e., milks) that are abundant, available and cheap; avoids safety or ethical concerns that are often associated with animal or clinical samples; and leverages commercially‐available molecular standards (proteins and antibodies). Further, the A2 milks have been proposed to offer health benefits (compared to A1‐containing regular milks). We used commercially‐available immunoassays to validate that our ECL method discriminates A2 milks from regular milks based on differences in the levels of the A2‐ and A1‐β‐casein protein.

We believe that this work is significant for three reasons. First, compared to gold standard immunoassays, our direct ECL method is simple, rapid, comparatively inexpensive, and does not require sample processing steps to remove interferents. Second, our ECL method is proline‐selective because of proline's high luminescence and possible quenching reactions by other components or prolyl residue's spatial localization in sample, but our method is generic in the sense that the proline can be detected irrelevant to whether it is present as single amino acid, a prolyl residue of a peptide or protein, or embedded within a complex matrix (i.e., milk). This residue‐based detection selectivity can be contrasted with sequence‐dependent selectivity of an immunoassay. Third, while the A2/A1 milk example allowed validation of ECL method (using available immunoassays), the application of this proline‐specific ECL method should have broader applications given the importance of proline and prolyl residues in biology. Thus, we envision this ECL method could be used to detect proline‐rich proteins (e.g., gluten) throughout the supply chain, as well as provide a tool for studying their digestion, absorption, excretion and conversion into intermediates (e.g., peptide) that can activate immune responses.

## 4 Experimental Section

### Chemicals and Samples

The following chemicals were purchased from Sigma‐Aldrich (St. Louis, MO): tris(bipyridine)ruthenium(II) chloride (Ru(bpy)_3_Cl_2_, Ru_b3_^2+^), phosphate‐buffered saline, 20 amino acids including alanine, arginine, asparagine, aspartic acid, cysteine, glutamic acid, glutamine, glycine, histidine, isoleucine, leucine, lysine, methionine, phenylalanine, proline, serine, threonine, tryptophan, tyrosine, and valine.

The following samples were tested in this study: 20 amino acids (Sigma–Aldrich, USA), A1‐ and A2‐oligopeptide synthesized (Biomatik Corporation, Canada), A1‐ and A2‐β‐casein standards obtained in A1‐ and A2‐β‐casein ELISA assay kit (Biosensis, Australia), all milks purchased in the grocery store including three different brand regular liquid milks and three different brand A2 liquid milks, one regular power milk and one A2 powder milk.

### Synthesis of Peptides

The peptide synthesis was conducted by Biomatik Corporation (Ontario, Canada). The peptide consists of 9 residues in the region between residues 63 to 71 of β‐casein, and includes a single amino acid variation at position 67. The sequence of synthesized peptides is PGPI**H**NSLP for A1‐peptide and PGPI**P**NSLP for A2‐peptide. Figure S4 (Supporting Information) provides the mass spectra of these peptides.

### Fabrication of 3D Printed Device

As reported in the previous work,^[^ ^25^ ^]^ a 3D printed device was fabricated with a Mars 3 Pro 3D printer with the standard black resin (ELEGOO, Guangdong, China) and was then attached to a 25 mm × 25 mm low resistance (3–5 Ω) ITO‐coated glass substrate (MSE Supplies, AZ) as the working electrode using the light curing resin (Hernon Manufacturing, FL). To cast the agarose salt bridge, the solution containing 1% agarose in 1 m KCl was first heated and then pipetted into the central well. After the agarose solidified, 1 m KCl solution was added to submerge the salt bridge. The Ag/AgCl reference electrode (Pine Research, NC) was then inserted into the central well containing the salt bridge via the side opening. A separate custom connector was also 3D printed. This connector comprised a platinum wire as the counter electrode and spring connectors (Digi‐Key, MN) for ITO electrode connections. With the device fully assembled, both the inserted reference electrode and the counter electrode would be immersed in the 1 m KCl solution present in the central salt bridge well.

### Electrochemiluminescence Measurement

The 3D‐printed device was placed into a microplate reader (Molecular Devices, CA) and connected it to an electrochemical analyzer (CH Instruments Inc., TX). The luminescence from 4 wells was measured with a microplate reader (SpectraMax, M2, Molecular Devices, LLC, CA) and 4 electric output signals from a device were simultaneously measured using an electrochemical analyzer (CHI1040C, CH Instruments Inc.). Cyclic voltammetry was performed for the ECL measurement by oscillating input potential between +0.4 V and +1.5 V at the scan rate of 10 mV s^−1^. A chronocoulometric measurement was performed by applying the step potential of +1.5 V for 2 min and then +0.4 V for 2 min.

### Chemiluminescence Image

The 3D‐printed device was placed into Amersham Imager 680 (GE life Science, UK) and connected it to an electrochemical analyzer (CHI1040C). A chronocoulometric measurement was performed by applying the step potential of +1.5 V for 2 min and then +0.4 V for 2 min. Chemiluminescence images (exposure time: 1s) were taken 10 s after switching oxidation (+1.5 V) or reduction (+0.4 V) potentials using Amersham Imager 680.

### ELISA Assay

To measure the amount of A1‐ and A2‐ β‐casein, a bovine A1 β‐casein and a A2 β‐casein sandwich ELISA assays (Biosensis, Australia) were used. This kit consists of β‐casein standards, a pre‐coated rabbit anti‐bovine β‐Casein polyclonal capture antibody, a chicken anti‐bovine A1 or A2 β‐Casein detection antibodies and a horseradish peroxidase (HRP)‐conjugated donkey anti‐chicken IgY antibody. The addition of a substrate (3,3′,5,5′ ‐tetramethylbenzidine, TMB) yields a colored reaction product that was directly proportional to the concentration of Bovine A1 or A2 β‐Casein present in milk samples and protein standards. Also, the protein standards in these ELISA kits were used to compare the difference of ECL responses between A1‐ and A2‐ β‐casein.

### Colorimetric Proline Assay

A ninhydrin‐based colorimetric proline assay (MSE Supplies, AZ) was employed to compare the ECL assay for detecting proline contents in samples. We prepared sample solution including 1 mm histidine and proline, 2 mm A1‐ and A2‐peptide, and two‐fold diluted milk using PBS. The prepared sample solutions were mixed with ninhydrin solution and the resulting solutions were incubated for 30 min in a heat block of 95 °C, and then the absorbance was measured at 470 nm.

### Statistical Analysis

Statistical analyses were performed using the software package R (R version 4.1). No pre‐processing of data was carried out for the stastical analyses. A cluster analysis was performed using K‐means method with the package of “factoextra”. The number of clusters was determined by silhouette method. Kruskal–Wallis test was used to compare two groups for continuous variables. The *p*‐value < 0.05 was considered statistically significant. Spearman's correlation coefficients were calculated to examine the correlation between two variables. The slope differences of cross‐modal plot were analyzed using a multiple linear regression model.

## Conflict of Interest

The authors declare no conflict of interest.

## Author Contributions

E.K. and G.F.P. conceptualized the study. E.K. developed the methodology and performed all experiments. C.C. designed and fabricated a 3D‐printed 4 well device. E.K., C.C., and M.J.C. performed the chemiluminescence imaging measurements. E.K. and M.H. analyzed the data. E.K., W.E.B, and G.F.P. interpreted the results. E.K. and G.F.P wrote the paper. All authors revised and approved the paper. G.F.P. supervised the project.

## Supporting information



Supporting Information

Supplemental Movie 1

Supplemental Movie 2

## Data Availability

The data that support the findings of this study are available from the corresponding author upon reasonable request.
