# Supplementary material for: Proline‐Selective Electrochemiluminescence Detecting a Single Amino Acid Variation Between A1 and A2 β‐Casein Containing Milks
Source: Adv Sci (Weinh). 2024 Dec 7;12(5):2411956. doi: 10.1002/advs.202411956 (PMC11792022; doi:10.1002/advs.202411956)
Supplement: Supplementary file 1 — Supporting Information [file ADVS-12-2411956-s002.pdf]

ADVANCED SCIENCE
Open Access

# Supporting Information

for *Adv. Sci.*, DOI 10.1002/advs.202411956

Proline-Selective Electrochemiluminescence Detecting a Single Amino Acid Variation Between A1 and A2 $\beta$-Casein Containing Milks

*Eunkyoung Kim*, Chen-Yu Chen, Monica J. Chu, Mya F. Hamstra, William E. Bentley and Gregory F. Payne**

# Supporting Information

Proline-Selective Electrochemiluminescence:
Detecting a Single Amino Acid Variation Between A1
and A2 β-Casein Containing Milks

*Eunkyoung Kim*, Chen-Yu Chen, Monica Joy Chu, Mya Hamstra,*
*William E. Bentley, Gregory F. Payne**

# Figure S1

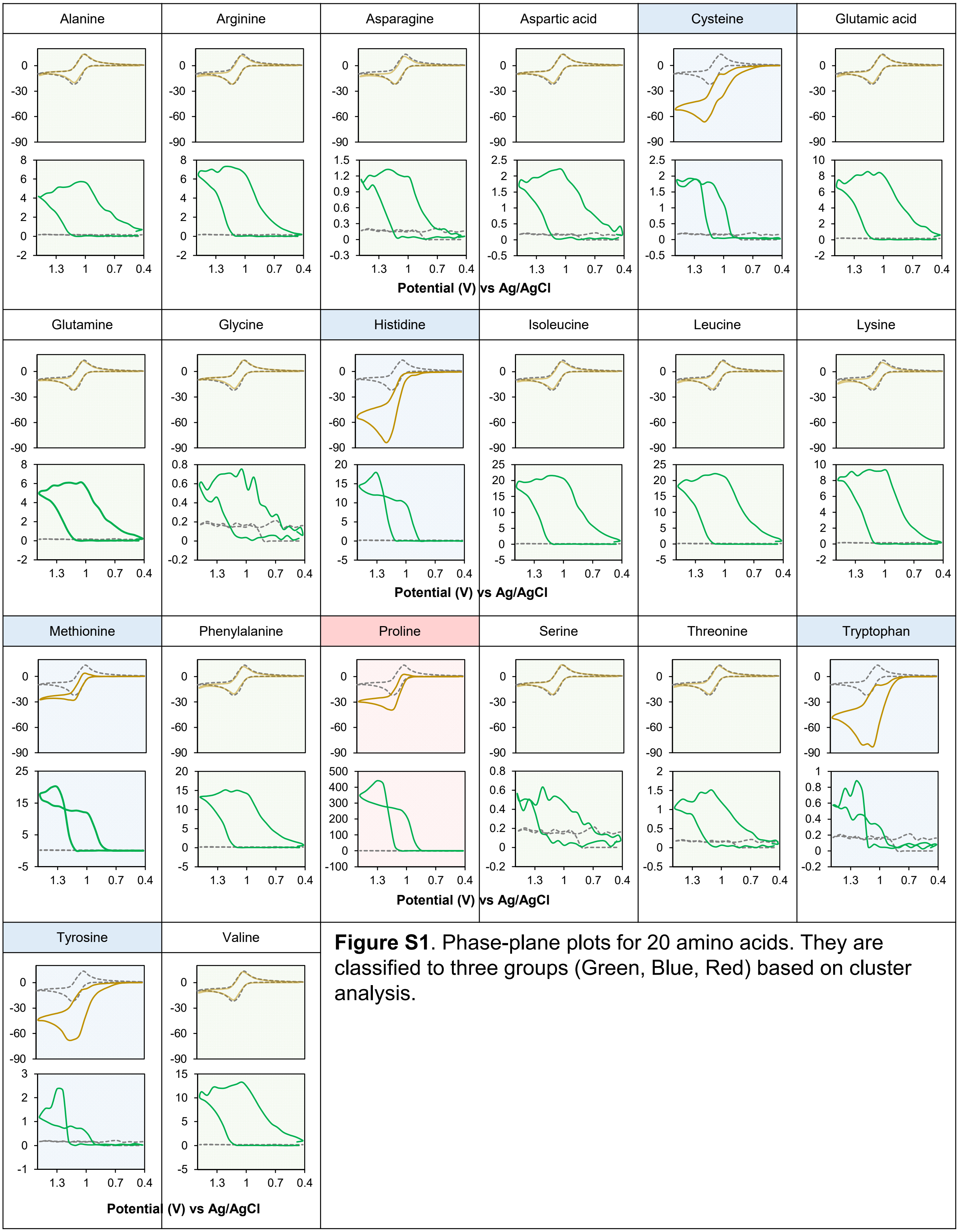


**Figure S1**. Phase-plane plots for 20 amino acids. They are classified to three groups (Green, Blue, Red) based on cluster analysis.

# Figure S2

| Amino acids | Relative ECL | |
|---|---|---|
| | *Anal.Chem.* 1992 | **This study** |
| PRO | 1 | 1 |
| LEU | 0.222 | 0.08138 |
| PHE | 0.1 | 0.049518 |
| VAL | 0.16 | 0.048183 |
| MET | 0.009 | 0.047611 |
| HIS | 0.013 | 0.041407 |
| GLU | 0.133 | 0.032905 |
| ARG | 0.044 | 0.023485 |
| ALA | 0.062 | 0.023326 |
| CYS | 0.012 | 0.006526 |
| THR | 0.007 | 0.004974 |
| ASN | 0.01 | 0.004221 |
| GLY | 0.009 | 0.002589 |
| SER | 0.004 | 0.002146 |
| TYR | 0.011 | 0.001533 |

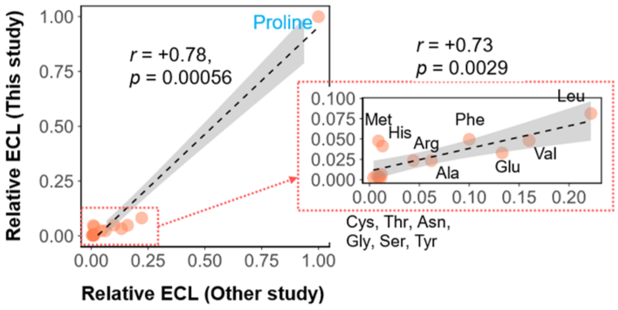


**Figure S2**. Comparison of our relative ECL responses for 15 amino acids with other measurement (*Anal.Chem.* 1992, 64, 166-170).

# Figure S3

(a)

| | Amino acids | Elect / $Q_{Tot}$ (mC) | Opt / $AUC_{Tot} \times 10^{-5}$ | Elect/ Log ($Q_{Tot}$) | Opt /Log ($AUC_{Tot}$) |
|---|---|---|---|---|---|
| 1 | Alanine | 2.66975 | 30.79801 | 0.426471 | 1.488523 |
| 2 | Arginine | 3.56475 | 31.0078 | 0.552029 | 1.491471 |
| 3 | Asparagine | 3.9185 | 5.573195 | 0.59312 | 0.746104 |
| 4 | Aspartic Acid | 2.61525 | 11.14194 | 0.417513 | 1.046961 |
| 5 | Cysteine | 21.3225 | 8.616888 | 1.328838 | 0.93535 |
| 6 | Glutamic Acid | 2.93675 | 43.44501 | 0.467867 | 1.63794 |
| 7 | Glutamine | 3.40875 | 26.32128 | 0.532595 | 1.420307 |
| 8 | Glysine | 2.57725 | 3.418932 | 0.411157 | 0.53389 |
| 9 | Histidine | 21.19 | 54.66991 | 1.326131 | 1.737748 |
| 10 | Isoleucine | 3.466 | 88.27679 | 0.539829 | 1.945847 |
| 11 | Leucine | 3.1925 | 107.4481 | 0.504131 | 2.031199 |
| 12 | Lysine | 3.84275 | 40.90702 | 0.584642 | 1.611798 |
| 13 | Methionine | 9.53075 | 62.86146 | 0.979127 | 1.798384 |
| 14 | Phenylalanine | 3.56625 | 65.38005 | 0.552212 | 1.815445 |
| 15 | Proline | 11.17 | 1320.321 | 1.048053 | 3.12068 |
| 16 | Serine | 2.89775 | 2.833147 | 0.462061 | 0.452269 |
| 17 | Threonine | 3.4395 | 6.567143 | 0.536495 | 0.817376 |
| 18 | Tryptophan | 11.1775 | 1.214072 | 1.048345 | 0.084244 |
| 19 | Tyrosine | 12.17 | 2.023699 | 1.085291 | 0.306146 |
| 20 | Valine | 3.25725 | 63.6176 | 0.512851 | 1.803577 |

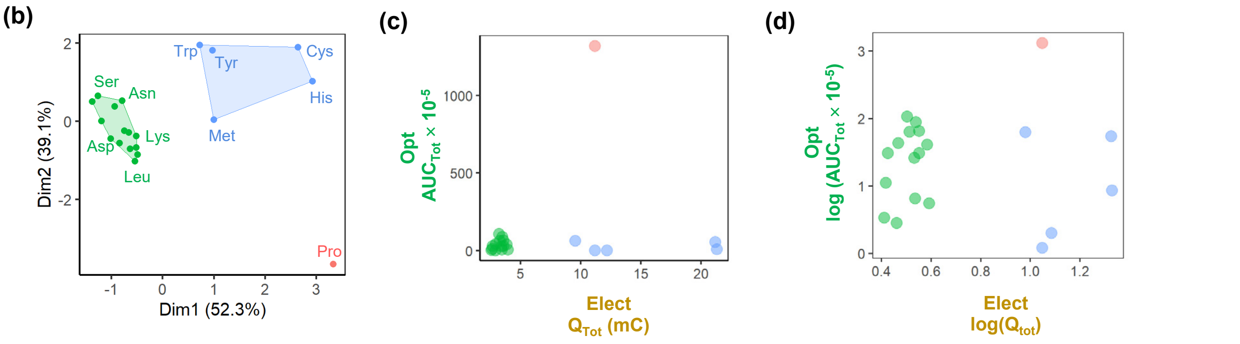


**Figure S3**. (a) Table of the electric and optical analyzed values for 20 amino acids. (b) Cluster analysis for 20 amino acids. (c) Cross-modal plot of electric and optical responses. (d) Cross-modal plot of logarithmic scales of electric and optical responses

# Figure S4

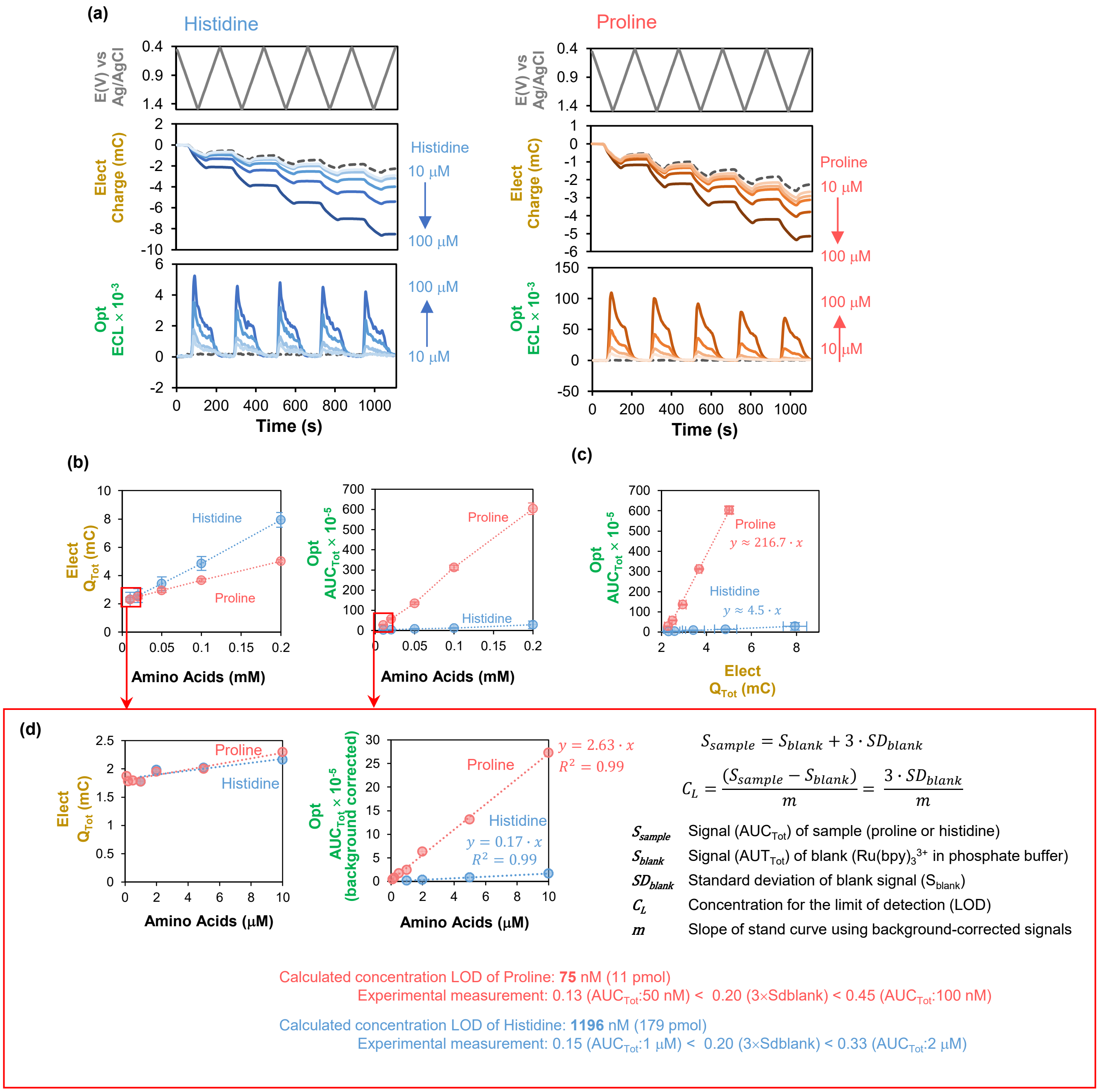


**Figure S4**. (a) Time series output plots for various histidine and proline (10 μM ~ 100 μM). (b) Electrical and optical responses linearly vary with amino acid concentration (0.01 mM ~ 0.2 mM). (c) Cross-modal analysis for various His and Pro. (d) Electrical and optical responses linearly increase even in the lower concentration region of amino acid (0.1 ~ 10 μM). From the optical response standard curve, the detection of limit (LOD) of histidine and proline were calculated based on the IUPAC Definition (Gary L. Long and J. D. Winefordner, "Limit of Detection A Closer Look at the IUPAC Definition", *Analytical Chemistry* **1983** *55* (07), 712A-724A, DOI:10.1021/ac00258a724). Experimentally, the LOD for proline was approximately determined to be 100 nM and for histidine to be 2 μM.

## Figure S5

A1-peptide: PGPI**H**NSLP        Molecular weight of histidine: 155.16 g/mol

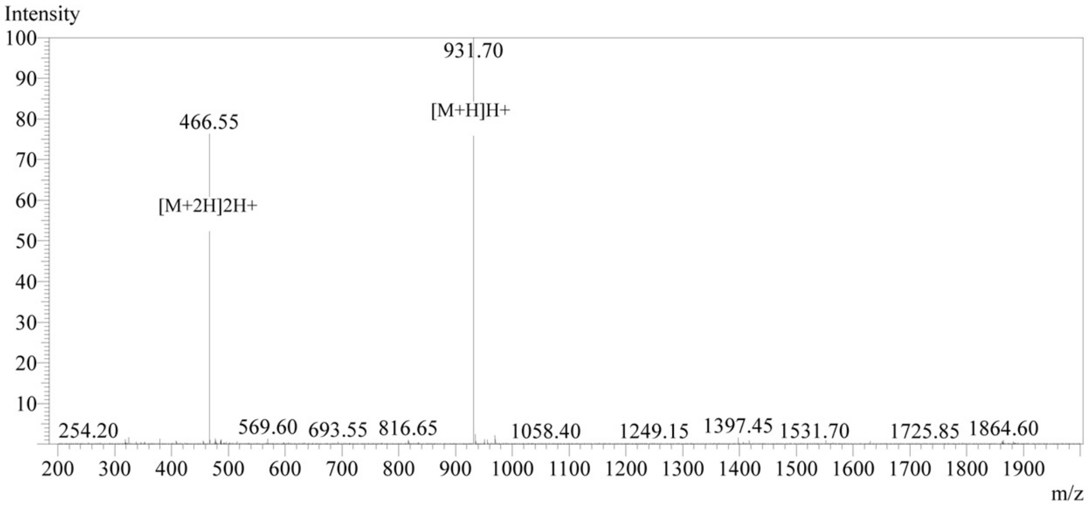


A2-peptide: PGPI**P**NSLP        Molecular weight of proline: 115.13 g/mol

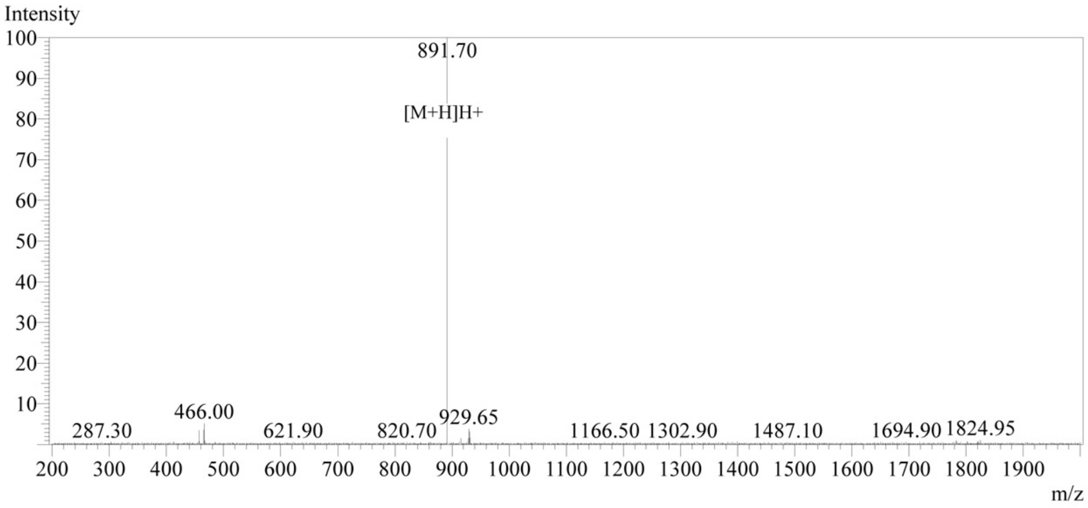


**Figure S5**. Mass spectrum of synthesized A1- and A2-peptides provided by Biomatik Corporation (Ontario, Canada). The molecular weight difference (931.70 – 891.70 = 40 g) of two peptides is consistent with the molecular weight difference (155.16 (His) – 115.16 (Pro)= 40 g) between histidine and proline.

# Figure S6

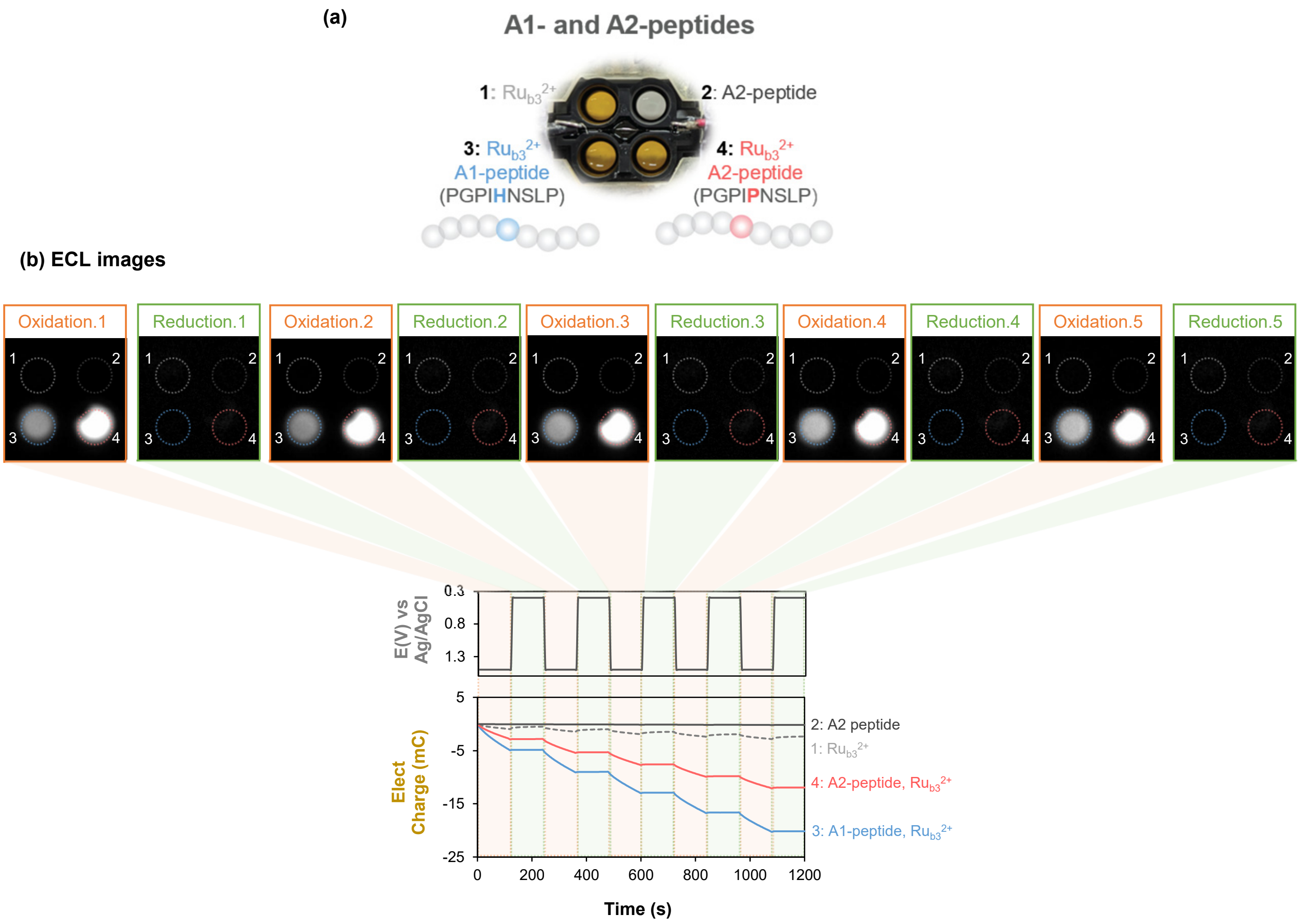


**Figure S6**. (a) Picture of 3-D printed 4-well device containing a different sample in each well. (b) ECL images taken when the oxidation (+1.5 V) and reduction (+0.4 V) potential are applied for 2 min. Imposed electrical step potential inputs and time-series electrical outputs.

# Figure S7

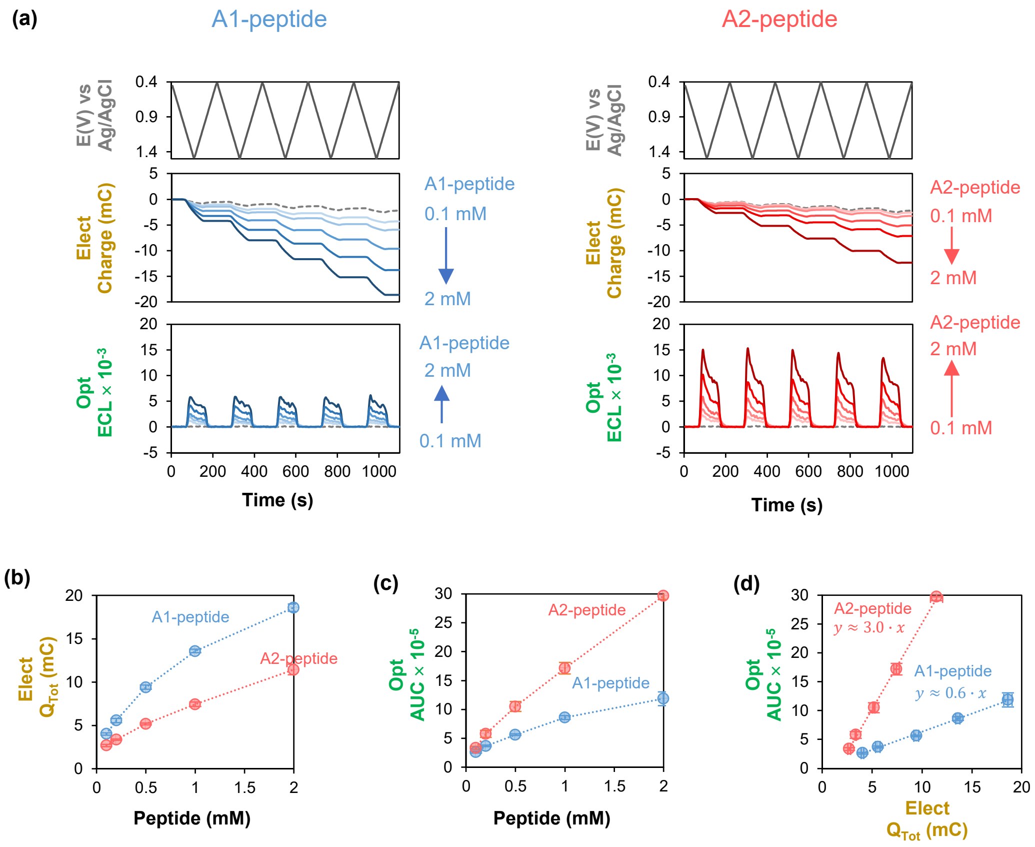


**Figure S7**. Time series output plots for various A1- and A2-peptides (0.1 mM ~ 2 mM). (b) Electrical and (c) optical responses for various peptide concentrations. (d) Cross-modal analysis for the A2 and A1 peptides.

# Figure S8

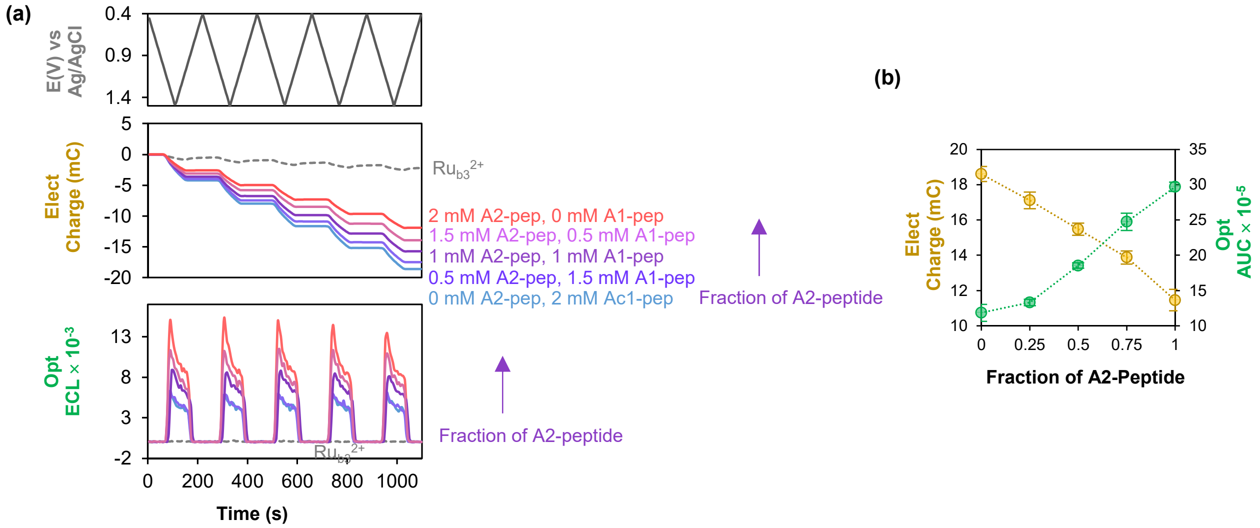


**Figure S8**. (a) Time series output plots for various fraction of A2-peptide in a mixed solution of A1- and A2- peptides. (b) Electrical and optical analyzed results dependent on the fraction of A2-peptide

## Figure S9

### Nutrition facts of liquid milk as provided on the milk's label

| | Regular milk (Reg.Milk) | | | A2 milk | | |
|---|---|---|---|---|---|---|
| | RM.1 | RM.2 | RM.3 | A2.1 | A2.2 | A2.3 |
| Brand | Weis | Walmart | Giant | A2 | Alexandre | Giant |
| Serving Size (ml) | 240 | 240 | 240 | 240 | 240 | 240 |
| Protein (g) | 8 | 8 | 8 | 8 | 9 | 8 |
| Fat (g) | 8 | 8 | 8 | 9 | 10 | 9 |
| Cholesterol (mg) | 25 | 35 | 35 | 35 | 30 | 35 |
| Carbohydrate (g) | 12 | 13 | 12 | 13 | 12.2 | 11 |
| Vit. A (mcg) | 110 | *N.A* | 110 | 114 | *N.A.* | 110 |
| Vit. D (mcg) | 2.5 | 2 | 2.5 | 2.5 | 2.5 | 3 |
| Calcium (mg) | 271 | 300 | 300 | 300 | 270 | 290 |
| Sodium (mg) | 105 | 130 | 125 | 130 | 76 | 120 |
| Potassium (mg) | 317 | 400 | 400 | 400 | 450 | 370 |

# Figure S10

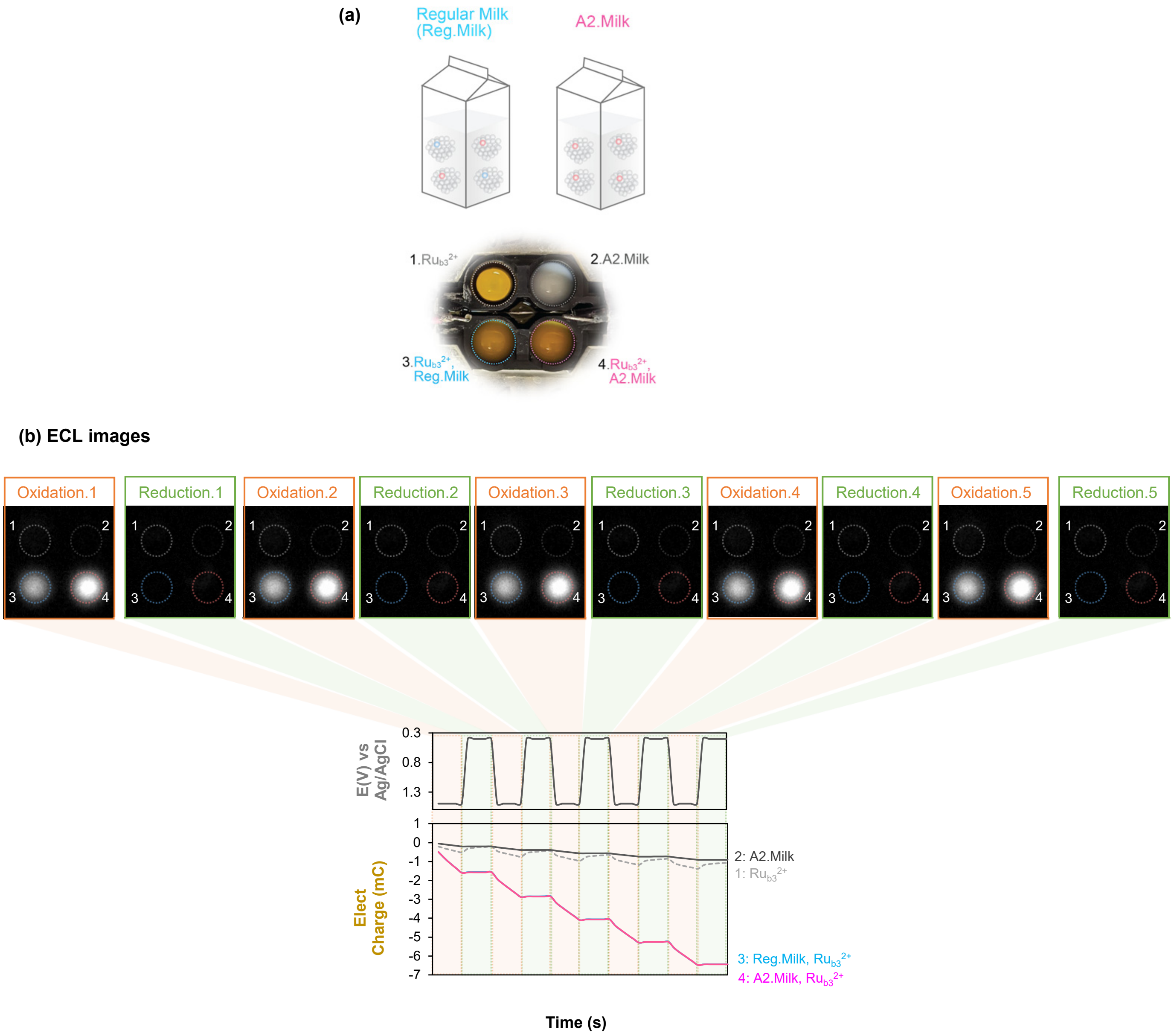


**Figure S10**. (a) Picture of 3-D printed 4-well device containing a different sample in each well. (b) ECL images taken when the oxidation (+1.5 V) and reduction (+0.4 V) potential are applied for 2 min. Imposed electrical step potential inputs and time-series electrical outputs.

# Figure S11

(a) **Nutrition facts for powder milks as provided on the milk's label**

| | Regular milk (Reg.Milk) | A2 milk |
|---|---|---|
| Brand | Hoosier Hill Farm | Alexandre |
| Serving Size (g) | 7 | 33 |
| Protein (g) | 2 | 9 |
| Fat (g) | 2 | 9 |
| Cholesterol (mg) | 5 | 30 |
| Carbohydrate (g) | 3 | 11 |
| Vit. A (mcg) | 5 | NA |
| Vit. D (mcg) | 0 | 0 |
| Calcium (mg) | 64 | 300 |
| Sodium (mg) | 25 | 90 |
| Potassium (mg) | 93 | 330 |

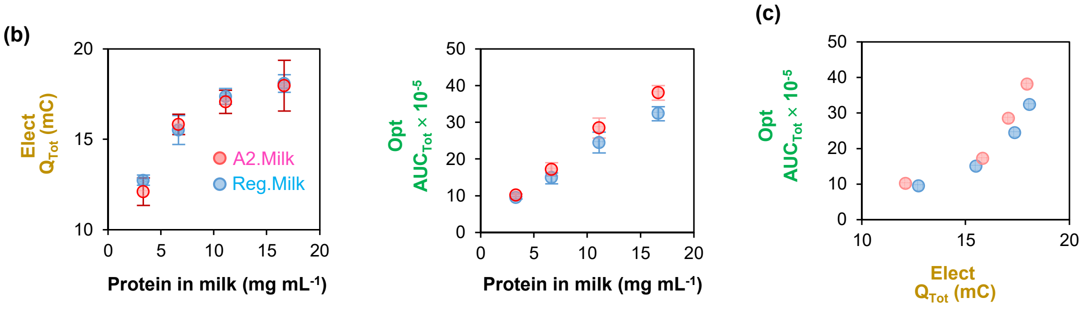


**Figure S11**. (a) Nutrition facts for regular and A2 powder milk as provided on the milk's label. (b) Electrical and optical responses and (c) cross-modal analysis for the regular and A2 powder milks dependent on various diluted powder milk .

# Figure S12

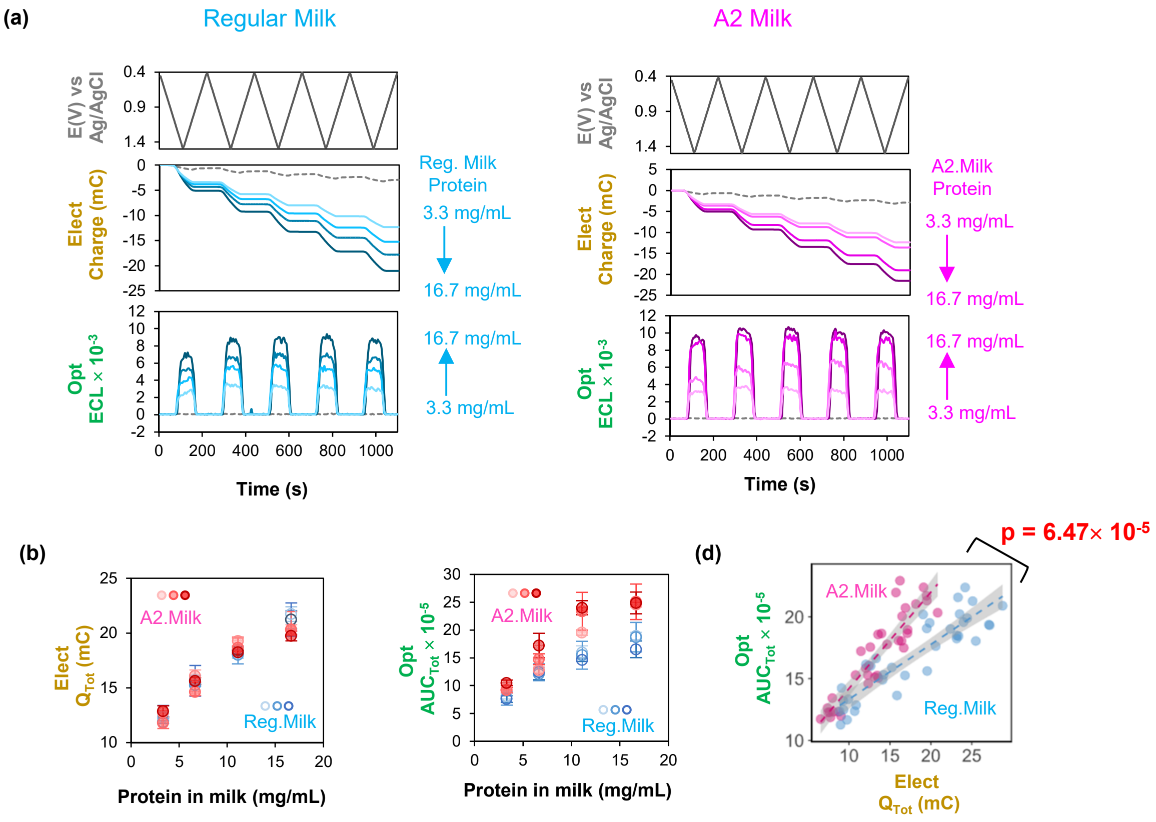


(e) lm(formula = Opt ~ Milk * Elect, data = D.Milk)

| | Estimate | Standard Error | P-value |
|---|---|---|---|
| (Intercept) | -4.7177 | 1.9929 | 0.0208* |
| MilkA2 | -7.7553 | 2.9365 | 0.0102* |
| Elect | 1.0755 | 0.1159 | $1.06\times10^{-13}$*** |
| **MilkA2:Elect** | **0.7287** | **0.1711** | **$6.47\times 10^{-5}$***** |

**Figure S12**. (a) Time series output plots for various diluted regular milk and A2 milk. (b) Electrical and optical responses vary with milk protein concentration. (d) Cross-modal analysis for the regular and A2 milks. (e) Statistical analysis of cross-modal plot using a multiple linear regression model to investigate the statistical significance of the slope differences between regular and A2 milk.

## Figure S13

| 10×dil Milk (3.3 mg/mL protein in milk) | 5×dil Milk (6.7 mg/mL protein in milk) | 3×dil Milk (11.1 mg/mL protein in milk) | 2×dil Milk (16.7 mg/mL protein in milk) |
|---|---|---|---|
| $p = 6.7\times10^{-4}$ | $p = 9.1\times10^{-3}$ | $p = 3.5\times10^{-4}$ | $p = 3.5\times10^{-4}$ |
| 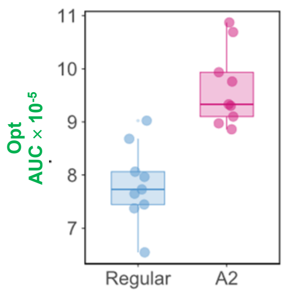  | 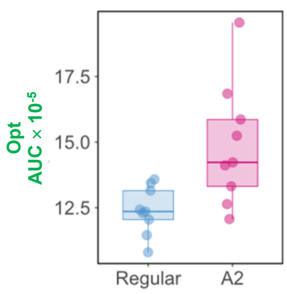  | 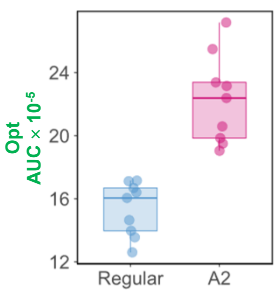  | 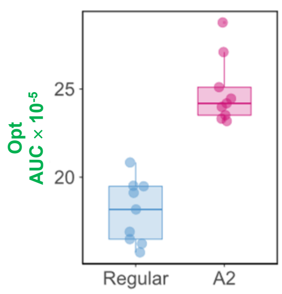  |

**Figure S13**. Consistently, optical responses of A2 milk are higher than those of regular milk in the different range of milk protein concentration (3.3 mg/mL ~ 33 mg/mL). All optical differences are statistically significant. The detection of limit (LOD) for milk protein to discern A2 from regular milk is estimated to ~ 3.3 mg/mL (10-fold diluted milk).

# Figure S14

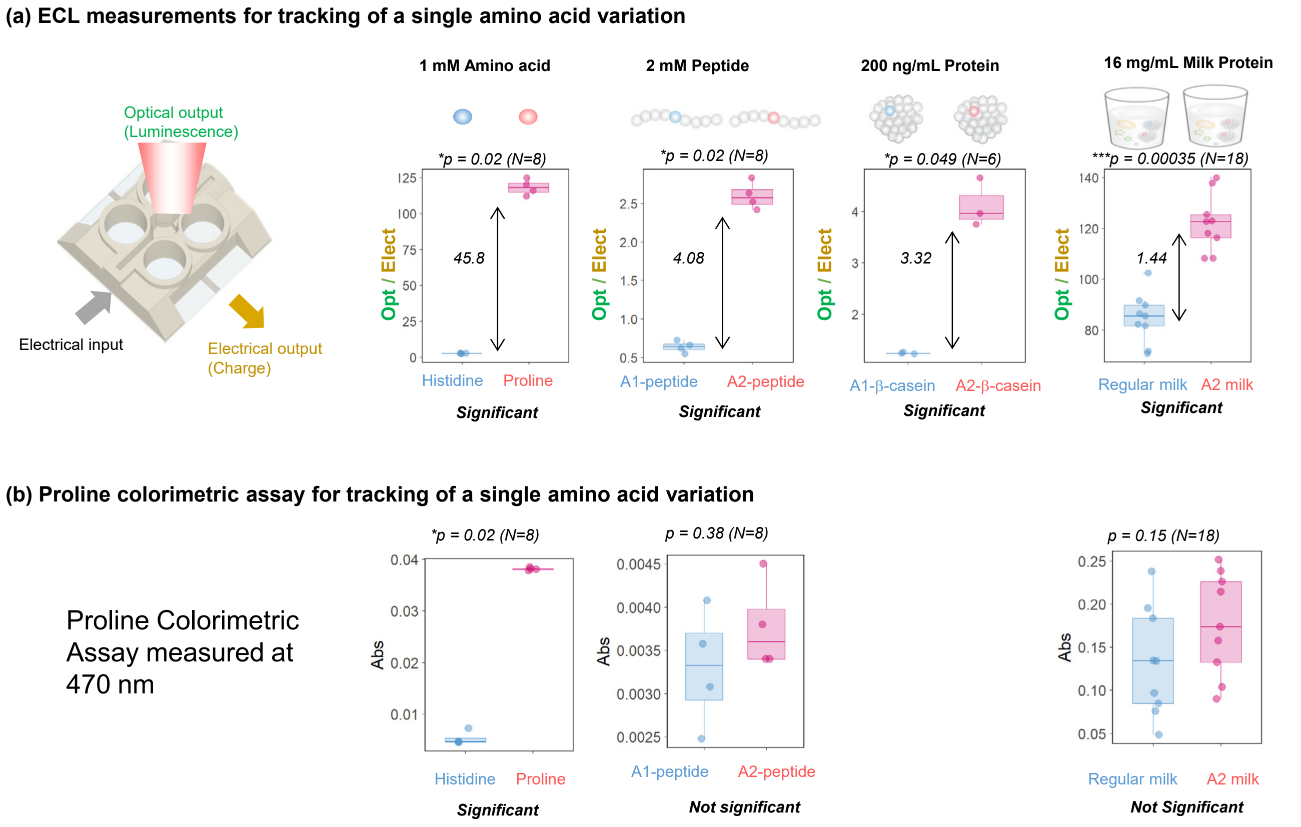


**Figure S14**. (a) The generic nature of the ECL method is illustrated by the cross-modal metric ($AUC_{Tot}$ / $Q_{Tot}$): this metric decreases as the analysis becomes more challenging (from distinguishing amino acids, to distinguishing proline-rich peptides and proteins, and samples in complex backgrounds) yet the ECL method can discern the A2 and regular milks. (b) A commercial proline colorimetric assay (MSE Supplies, AZ) can discern proline from histidine but cannot distinguish a single amino acid variation in peptides and milks.
